# IDMA-Based MAC Protocol for Satellite Networks with Consideration on Channel Quality

**DOI:** 10.1155/2014/181734

**Published:** 2014-07-13

**Authors:** Gongliang Liu, Xinrui Fang, Wenjing Kang

**Affiliations:** School of Information and Electrical Engineering, Harbin Institute of Technology, No. 2 West Wenhua Road, Weihai 264209, China

## Abstract

In order to overcome the shortcomings of existing medium access control (MAC) protocols based on TDMA or CDMA in satellite networks, interleave division multiple access (IDMA) technique is introduced into satellite communication networks. Therefore, a novel wide-band IDMA MAC protocol based on channel quality is proposed in this paper, consisting of a dynamic power allocation algorithm, a rate adaptation algorithm, and a call admission control (CAC) scheme. Firstly, the power allocation algorithm combining the technique of IDMA SINR-evolution and channel quality prediction is developed to guarantee high power efficiency even in terrible channel conditions. Secondly, the effective rate adaptation algorithm, based on accurate channel information per timeslot and by the means of rate degradation, can be realized. What is more, based on channel quality prediction, the CAC scheme, combining the new power allocation algorithm, rate scheduling, and buffering strategies together, is proposed for the emerging IDMA systems, which can support a variety of traffic types, and offering quality of service (QoS) requirements corresponding to different priority levels. Simulation results show that the new wide-band IDMA MAC protocol can make accurate estimation of available resource considering the effect of multiuser detection (MUD) and QoS requirements of multimedia traffic, leading to low outage probability as well as high overall system throughput.

## 1. Introduction

Compared to a narrow-band system, a wide-band system [1] can support much higher traffic rates and provide satisfactory multimedia services. Broadband satellite network is a typical system with limited bandwidth and power. How to effectively utilize the precious communication resources while ensuring the requirements for quality of all kinds of services at the same time is thus an urgent problem to be solved.

Multiple access control (MAC), which provides a mechanism of sharing the satellite resource efficiently, plays a vital role in enhancing the utilization of radio resource. In order to effectively optimize the allocation of resources and the onboard power, a new medium access control (MAC) techniques should be proposed. Besides, the core of the MAC technology is multiple access schemes, which can coordinate users sharing the limited resources to achieve efficient and reliable transmission.

In most of the existing broadband satellite communication systems, it is feasible to use frequency division multiple access (FDMA) or time division multiple access (TDMA). However, there exist some technical bottlenecks, particularly in the frequency reuse aspect and system capacity.

The system of code-division multiple access (CDMA) is attractive for its outstanding capacity, frequency-spectrum utilization, and reliability, while tremendous computational cost on multiuser detection (MUD) to eliminate multiple access interference (MAI) is needed in this system.

Interleave-division multiple access (IDMA), derived from CDMA [2], provides a new solution for multiple access in satellite networks. As in the latest proposed multiple access scheme, the key thought of IDMA is to use different interleavers to distinguish multiple users, in which way users can transmit their information simultaneously. Consequently, the QoS is satisfied without the utilization of complicated slot management or packet scheduling which are both necessary in TDMA or CDMA systems, leading to the reduction in the complexity of onboard queuing and switching. Furthermore, IDMA adopts the iterative chip-by-chip (CBC) detection scheme to combat both intercell and intracell multiple access interference (MAI). Compared to CDMA, IDMA solves the problem of MAI at a lower computational complexity, the decrement of which is linear with the number of users [2].

The studies in [3] forecast and modify the ground mobile environment with the time-series model such as glide average, exponential smoothness, and linearity regress. Considering that the satellite channel is different from wireless channels on the ground, channel prediction model of ground system cannot completely be suitable for satellite channel. Meanwhile, due to the long-time delay and nonstationary of satellite channel, the predictable length of most prediction algorithm is limited by the relative time of the input sequence. In this case, prediction data for controlling the rate adaptation and power allocation strategy is out of date, and therefore it is impossible to achieve real-time resource allocation. In [4], channel quality can be obtained directly by physical information such as signal-to-noise ratio (SNR), received signal strength, and bit error rate (BER). However, this method is rather complex and time consuming especially under bad channel condition. In this paper, ARIMI model based on smoothing processing and force feedback is proposed, achieving a relatively accurate prediction with the simple implementation structure.

In this paper, we introduce a new MAC protocol for wide-band IDMA satellite communication systems. Firstly, we develop a new IDMA-based power allocation algorithm combined with the technique of SINR evolution and channelprediction to provide good quality of service to as many mobiles as possible, even for the users under poor channel conditions. In [5], a study on a minimum-power allocation for multimedia traffic focuses on minimizing the received interference in wide-band CDMA networks. However, the optimum power allocation cannot be obtained due to ignoring the efficiency of MUD. A novel power allocation algorithm [6] combining the technique of SINR evolution and load balance can be accurately estimated when channel is assumed perfect, leading to low outage probability. Whereas the transmission environment may change sharply over time, the allocation of resources is inaccurate and unequal in the current. In a word, the new power allocation considered here is jointly based on the SINR evolution and variable channel quality, which is different from that in [6].

By taking the satellite channel prediction and adaptive transmission technique into account, the rate adaptation of multimedia can be achieved dynamically according to the satellite feedback control. And in order to improve system performance and capacity further, a new admission control mechanism based on channel quality and rate adaptation is proposed for IDMA system considering the effect of MUD. Rate adaptation algorithm ensures dynamic resource allocation by considering the physical and MAC layer jointly, which optimizes the system performance. In addition, considering the sufferable delay of handoff and new calls, the strategy of buffer queue for new calls [7] is also introduced to improve the blocking probability. Although most of the previous call admission control schemes combine the rate adaptation strategy and buffer queue strategy together to improve the end-to-end performance, very few of them consider the validity for buffering the new calls due to strict delay requirement and fairness of different users. What is more, the effect of multiuser detection on the performance of CAC algorithm is also evaluated in this paper.

The rest of this paper is organized as follows. In Section 2, the overall MAC protocol is described. In Section 3, the satellite channel prediction is illustrated. The dynamic power allocation algorithm is derived for wide-band IDMA system in Section 4. Based on the result of channel prediction, the multimedia wide-band IDMA rate scheduling scheme is developed in Section 5, and the effective CAC scheme is derived in Section 6. In Section 7, performance of the new MAC protocol is evaluated through simulation. Finally, the conclusion of this paper is drawn in Section 8.

## 2. New Mac Protocol

### 2.1. System Model

The wide-band IDMA system has the following features [8].

- No complex frame synchronization process: as different users occupy different interleavers in the process of transmission, the IDMA system supports asynchronous transfer mode (ATM) and does not need complex frame synchronization process. Thus, for each user without complex transmission scheduling, their QoS requirements can be satisfied easily.
- IDMA-CBC MUD and SINR evolution technique: considering the effects of the detector signal-to-noise ratio on MUD efficiency, resource allocation on board can be estimated accurately. Thus, based on the semianalytical SINR evolution technique, MUD efficiency is considered here as the percentage of the intracell interference cancelled by the multiuser detector, and the efficiency fully reflects the performance advantages of IDMA system.
- Interleave-division S-ALOHA access mechanism: compared to the traditional S-ALOHA, the interleave-division S-ALOHA (IDSA) access mechanism can improve the efficiency of the random access and shorten the access delay effectively. Considering that different interleavers are used to distinguish the access requests of multiple users in IDMA, we can balance the allocated access interleavers with traffic load interleavers to make a tradeoff between system performance and onboard processing complexity.
- Variable-rate transmission and variable processing gain: the IDMA system saves the bandwidth which is occupied for spread coding in the conventional CDMA system for channel coding. Thus, the coding gain maximization can be achieved by low bit-rate coding. For the IDMA system, the processing gain is determined by two factors: FEC coding gain and spreading gain. Considering that the greater coding gain can improve the BER performance of IDMA system in the physical layer, the demanding rate can be well adjusted by the FEC coding rate to meet the needs of various users. In that case, this paper uses a variable processing gain IDMA (VSG IDMA) model.

**Figure 1:**
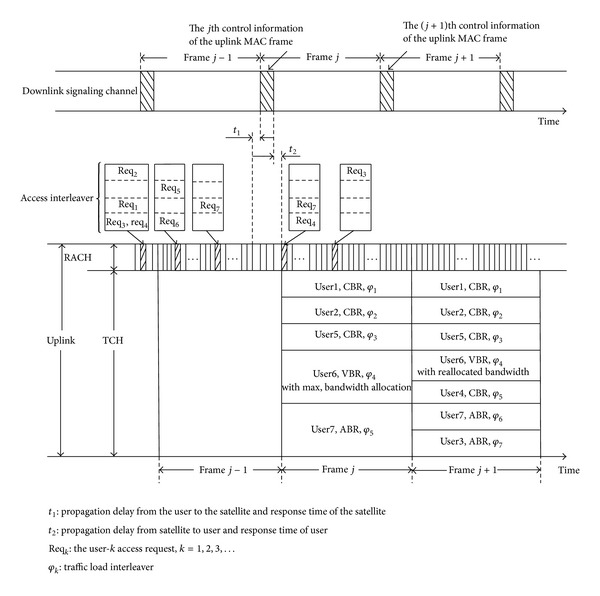
MAC protocol of IDMA.

In Figure 1, the schematic shows the MAC protocol of IDMA system. The uplink physical channel is divided into a random access channel (RACH) to send requests from a mobile terminal to the satellite repeater and a traffic channel (TCH) to send feedback of resource allocation from satellite repeater to mobile terminals by different interleavers. Ground station, via the RACH, sends requests to the onboard processor which makes decisions according to the current system load, service level, channel quality, and so on. Meanwhile, onboard processor needs to allocate channel resources (interleaver) and suitable power to the ground station.

Access request needs to include the following information: the coding type, the supportable maximum transmitted power, service classes, QoS requirements in bit error rate, bandwidth, and so forth.

### 2.2. Procedures of the MAC Protocol

The mechanisms of the new wide-band IDMA MAC protocol based on channel quality in wireless multimedia networks are shown in Figure 1. Compared with other multiple access systems, IDMA may provide users with flexible multirate and multiQoS support by controlling coding rate and power. Thus, it integrates the factors of service types, service quality, coding scheme, and permissible power factor in the existing MAC protocols. What is more, an optimized resource allocation proposal integrated with link quality prediction, power control, and call admission control can ensure dynamic resource distribution and change adaptively according to the environmental variation.

**Figure 2:**
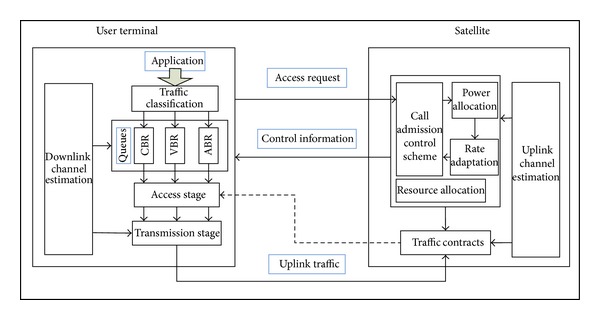
The framework of QoS guarantee mechanism.

As shown in Figure 2, QoS guarantee mechanism includes the following main functions [9].

- Channel quality prediction: the dynamic estimation of satellite channel provides the foundation and prerequisite for efficient resource allocation, particularly for resources such as bandwidth and power. Meanwhile, because of the long-transmission delay for satellites communication, the communication channel between the satellites and the mobile terminal constrains the performance of the whole system. Hence, timely measurement and accurate prediction on satellite channel are rather necessary to ensure effective resource allocation and adjustment.
- Power allocation algorithm: the performance of power control is the key to the quality of call admission control. The novel power allocation algorithm proposed combining the technique of SINR evolution and channel quality estimation can realize the optimized allocation of resources for each user and adjust the transmitted power adaptively according to the wireless environment. The algorithm not only illustrates the high efficiency of CBC MUD but also provides reliable communication for a growing number of users, especially when suffering the poor channel quality problem.
- Rate scheduling: in the transmission state, user sends a transmission request in RACH before it transmits a batch of packets. The wireless channel measurement results can be divided into superior channel quality and inferior channel quality. Users with inferior channel quality perform rating downgrades to maximize the throughput of the system. Rate scheduling strategies guarantee the priority of different services, meeting the requirement of service with maintainable bit rates.
- Call admission control: when communication is initiated, a mobile terminal sends an admission request in RACH with a randomly selected interleaver. When the satellite receives such a request, a new effective interference-based CAC algorithm is invoked to check if enough resources are available in the system. The result is sent back to the mobile terminal through BCH. If the answer is positive, the request is accepted and the interleaver is reserved for the mobile terminal. And then, the mobile terminal enters the transmission state. Otherwise, considering that handover calls own a higher priority than new calls, call admission policy will change with different types of calls. On one hand, because new calls are insensitive for time delay, the caching strategy of exponential backoff can efficiently decrease blocking probability. On the other hand, as for handover calls, the rate for the accepted user can be demoted further on the premise of fulfilling the user's requirements. Meanwhile, the new CAC can make accurate estimation of available resource considering the effect of MUD, leading to low outage probability as well as low blocking and dropping probability.
- Mapping between physical and MAC layer: a cross-layer resource allocation method for IDMA systems is proposed by considering jointly the physical and MAC layer, which optimizes the system performance. The adaptive resource allocation is not an isolated problem of access control or power allocation problem, but a global optimization one restrained by QoS and channel quality [10].

## 3. Satellite Channel Quality Prediction

With the properties of time-varying, multipath effect, shadow fading, and Doppler shift, satellite communication environment impacts the reliability of digital signal transmission and the effectiveness of onboard resource allocation. Thereby, the communication channel between the satellite and the mobile terminal constrains the performance of the whole system.

In order to effectively predict the change of the channel quality and assess the rationality of the resource allocation scheme, channel model should be established veritably to reflect the practical environment.

### 3.1. Model of the Satellite Communication System

Based on the transmission characteristics of satellite mobile communication channel, we present and simulate three common channel models of wide-band satellite mobile communication. The fading characteristics of the received signal, the level crossing rate (LCR), and average fade duration (AFD) for C. Loo, Corazza, and Lutz are simulated and analyzed. These important statistical properties drive the statistical regularities about fading speed and duration of the received signal, which provide efficiency and accuracy of theoretical support for the following research. The following provides a detailed example of Corazza model:

(1) $$\begin{array}{c} r\left(t\right)=\left[z\left(t\right)+d\left(t\right)\right]s\left(t\right)=R\left(t\right)s\left(t\right), \end{array}$$

where *s*(*t*) is in line with random process of the lognormal distribution, which is regarded as large scale fading and *z*(*t*) and *d*(*t*) represent light-of-sight (LOS) and multipath component, respectively.

Various parameters of the Corazza model with a least squares fitting method are deduced in the literature [11]. The main parameters studied were the coefficients of mathematical expectation *u*, variance *d*_0_ of ln *s*, and Rice factor *K* varied with satellite elevation. The parameters of the literature [11] apply to any elevation ranging from 20 to 80.

**Figure 3:**
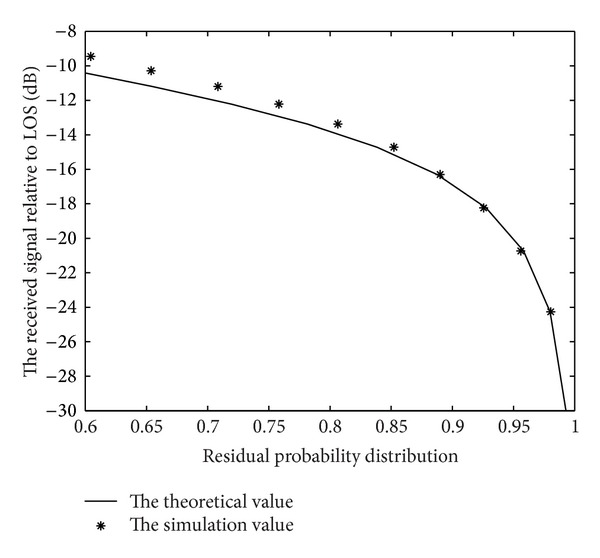
The cumulative probability curve of Corazza model.

The cumulative probability curve of the theoretical formula and the measured data were separately fitted at the speed of 60 km/h, in the countryside. With the satellite elevation equal to 20, the result is shown in Figure 3.

**Figure 4:**
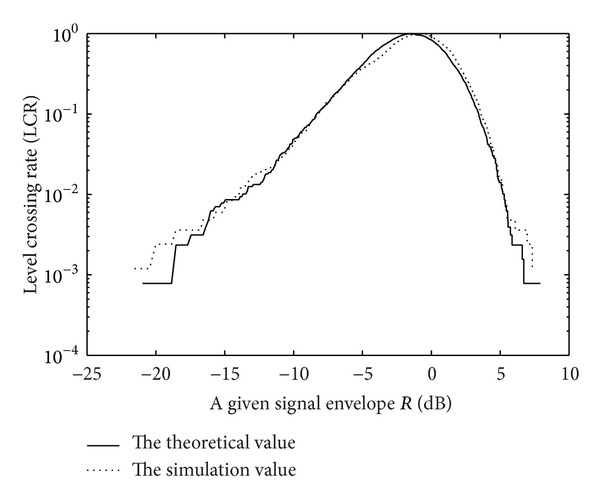
The level crossing rate of Corazza model.

**Figure 5:**
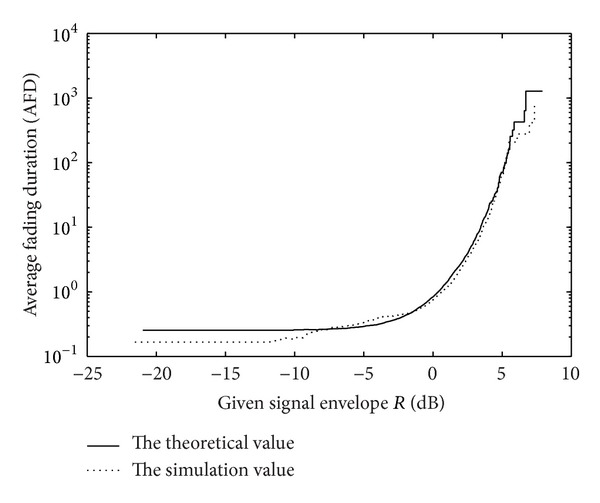
The average fades duration of Corazza model.

At the same time, the second order statistics which mean average level crossing rate as well as average fade duration of each composite fading channel are analyzed and, according to the literature [11], corresponding uniform expressions can be got. Simulation results on the fitting are shown in Figures 4 and 5.

Obviously, the channel influences the signals in two ways: the large scale fluctuation mainly caused by the shadow effect and the small scale fluctuation by multipath effect and Doppler shift. Indeed the occlusion caused by the terminal movement always remains a minor variation or constant over time, while rich multipath signal changes sharply with the mobile terminal. Therefore, the change of large scale attenuation rate is generally far below that of the small scale fading.

### 3.2. ARIMA Model for Satellite Channel Quality Prediction

The comparisons of signal containing small scale fading and that without the small scale fading under severe environments proved that fluctuation speed of the actual signal is mainly dominated by small scale fading in the literature [12]. Moreover, regardless of the impact of small scale fading, signal wave speed will be greatly reduced. The actual measurement of large scale fading signal and fitting model has been given in the literature [13]. The calculation results show that channel quality forecast is feasible and practical when the actual signal is replaced by the signal affected by large scale fading only.

Based on the above analysis, we choose ARIMA prediction channel to forecast the quality of satellite channel when removing the effect of small scale fading.

Supposing that TTI length is 1 ms, the actual satellite data received is a string sequence with 1 ms intervals. The SNR of downlink signal received by all mobile users can be able to directly indicate the channel quality. According to the literature related, the end-to-end delay from the ground station via satellite to the destination end (user), caused by double jump mode, is 540 ms. To reduce the prediction step length, it is feasible to adopt 100 TTI channel status to estimate a channel state, namely, 100 ms statistics for a state. Therefore, the prediction of channel quality by 540 ms requires just six steps of prediction, concluded in 500 ms to 600 ms time period.

**Figure 6:**
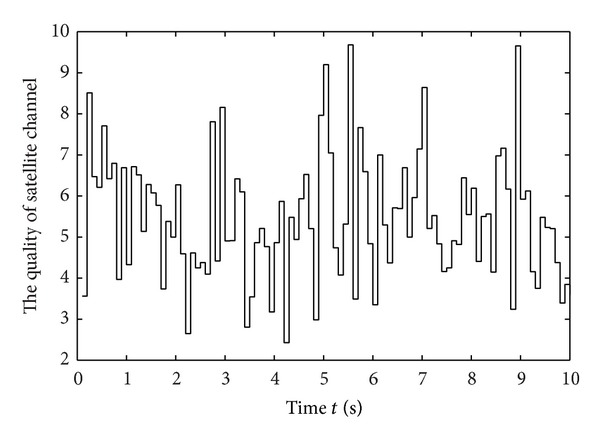
The state of channel quality.

As fluctuation values within a certain range were calculated as a single state, relative time of each state will be longer than that of the specific value. Then, reasonable resource allocation can be realized according to the predicted state of channel quality.  Figure 6 illustrates the measurement and quantitative data of satellite channel quality and depicts the rapid change of the channel quality.

**Figure 7:**
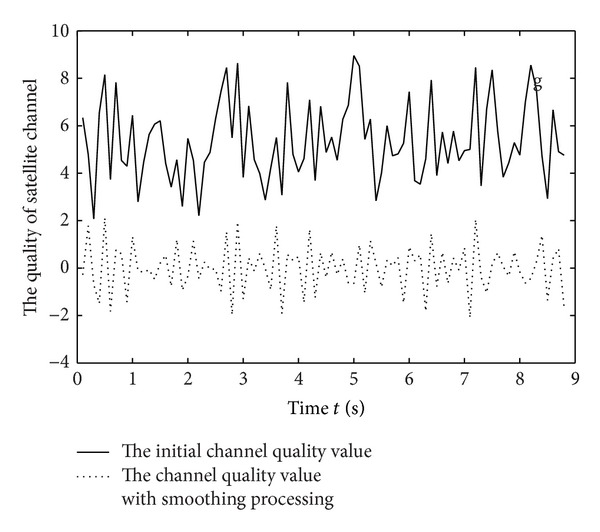
The prediction data of satellite channel quality with smoothing processing.

The prediction data of satellite channel quality with smoothing processing for many times can be seen from Figure 7. As is shown in Figure 7, the signal of eliminating the small scale fading effect is stable.

**Figure 8:**
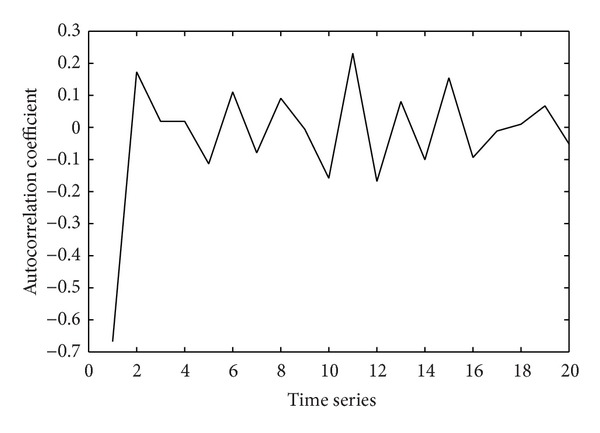
Autocorrelation analysis.

The autocorrelation for the prediction data of satellite channel quality with smoothing processing is shown in Figure 8. Because the correlations of signals are tending towards stability after operation many times, as can be seen, we can give prediction to the data by using the data correlation. Correlation time between states would be, in some sense, longer than that of the actual data. In other words, ARIMA model would obviously improve the precision of forecasting and increase the reliability of prediction with enhancement in temporal sequence correlation.

**Figure 9:**
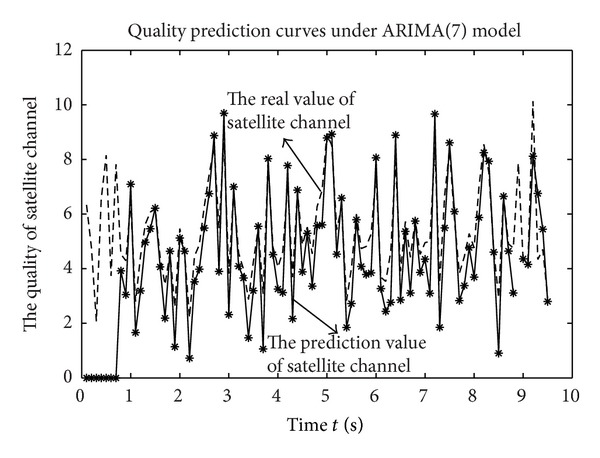
Comparison of channel quality sequence and data with smoothing processing.

As the data window of high order model is fairly large, the volume of historical data which is used to predict trend is increasingly large. Under the condition of the small window, we can develop low order model by introducing data retroactivity to approximate high order model.  Figure 9 illustrates the quality of satellite channel while utilizing the step length 90 to predict the step data length 96 shows a certain error, but it is sufficient for handling a pretty good precision.

## 4. A Dynamic Power Control Algorithm

### 4.1. IDMA-CBC MUD and SINR Evolution Technique

Combining with the specific IDMA-CBC MUD, we consider the capacity analysis further, which effectively resists the internal MAI. Complete structure and procedure of IDMA-CBC MUD are given in the literature [2].

With single path channel being synchronous and with modulation sett as BPSK in this study, the performance of IDMA-CBC detection scheme is mainly reflected by the decrease in the variable variance, for example, the variance of {*x*_*k*_(*j*), ∀*k*, *j*}. It can be written as

(2) $$\begin{array}{c} V_{k}=1-\tanh^{2}\left(\frac{Y_{{\text{SINR}}_{k}}}{2}\right),\;k=1,\ldots ,K. \end{array}$$

As shown in (2), *V*_*k*_, for example, the variance of an arbitrary chip from user-*k*, which is the corresponding power interference factor in the iteration, is the function of SINR_*k*_. Besides an anti-inference percentage with fixed SINR, the function *f*(SINR) is referred as the expectation of the interference power and written as

(3) f(SINRk)=E(Vk)=1−E[tanh2⁡(YSINRk2)],      k=1,…,K.

When the iteration reaches the iteration convergence point for user-*k*, equivalently, the system achieves maximum MAI elimination capacity; thus we define

(4) $$\begin{array}{c} {\text{SINR}}_{k}=\frac{P_{0}}{\sum_{i\neq k}^{}P_{i}E\left(V_{i}\right)+P_{N}}\geq \gamma_{k}, \end{array}$$

where *γ*_*k*_ represents (*E*_*b*_/*I*_0_)_req_ · (*R*_*b*_/*B*), and the total interference can be expressed as

(5) $$\begin{array}{c} I_{\text{total}}=\underset{i\neq k}{\sum }P_{i}f\left({\text{SINR}}_{i}\right)+P_{N}, \end{array}$$

where *f*(SINR_*i*_) is negatively correlated with SINR, ranging from 0 to 1, which has been verified in the literature [2].

### 4.2. Minimum-Power Allocation

Based on the variable spreading gain (VSG) of the program, multibit transmission rate can be got by adjusting the variable spreading gain. Therefore, in order to satisfy the QoS requirements of all users, we need to adjust the transmitted power of each user to minimize the interference to other users' further.

Assume that there exist *N*_*k*_ users for service type *k* supported in the multicell IDMA system, in which each user adopts the same spreading code and coding rate to transmit information. *W* is the spread-spectrum bandwidth; *R*_*i*_ is the data rate of the user *i* determined only by the spread gain SG; *P*_*i*_ and *h*_*i*_ represent the transmitted power and the uplink channel gain of the user *i*, respectively.

Considering the home cell, (*E*_*b*_/*I*_0_)_*k*_ of the special user *n*_*k*_ can be written as

(6) (EbI0)k=hnkPnk(Itotal−hnkPnk)·WRnk≥γk,      nk=1,2,…,Nk.

The constraints that the transmitted power and the data rate must fulfill are as follows:

(7) $$\begin{array}{c} 0<P_{n_{k}}<p_{i},\;R_{n_{k}}>r_{n_{k}},\;n_{k}=1,2,\ldots ,N_{k}, \end{array}$$

where *s*_*i*_ and *r*_*i*_ represent maximum permissible transmitted power and maintain data rate, respectively.

To adjust the transmission power of users, we add the general constraints to (6) and (7):

(8) Minimize∑nk=1NkPnk, nk=1,2,…,Nsubject to  0<Pnk<pi,    Rnk>rnk.

*Assumptions.*

1. QoS requirement of each user is equal to the target signal-to-noise ratio.
2. Data rate of each user is higher than the maintained data rate.

The optimal power value *P*_*n*_k__* obtained in the sense of the above assumptions is expressed as

(9) $$\begin{array}{c} \frac{h_{n_{k}}P_{n_{k}}^{*}}{\left(I_{\text{total}}-h_{n_{k}}P_{n_{k}}^{*}\right)}\cdot \frac{W}{R_{n_{k}}}=\gamma_{k},\;\forall n_{k}=1,\ldots ,N_{k}. \end{array}$$

The *I*_total_ in this case can be calculated as

(10) $$\begin{array}{c} I_{\text{total}}=h_{n_{k}}P_{n_{k}}^{*}\left(1+\frac{W}{\gamma_{k}R_{n_{k}}}\right). \end{array}$$

Considering the multicell IDMA system, the total interference power including the intracell interference from users *I*_intra_, the received power from adjacent cells *I*_inter_, and the thermal background noise *P*_*N*_ can be calculated as

(11) $$\begin{array}{c} I_{\text{total}}=I_{\text{intra}}+I_{\text{inter}}+P_{N}. \end{array}$$

According to the interference calculation model, the other-beam interference factor *f*_other_, which is defined as the ratio of the interference power received from the other beams *I*_inter_ to the interference power produced by users in local beam *I*_intra_, can be calculated as

(12) $$\begin{array}{c} f_{\text{other}}=\frac{I_{\text{inter}}}{I_{\text{intra}}}. \end{array}$$

As average interference is utilized, the other-beam interference factor *f*_other_ presented in previous studies [14] can be seen as a constant 0.55. Consequently, with (11) and (12), we can represent the *I*_total_ as

(13) $$\begin{array}{c} I_{\text{total}}=\left(1+f_{\text{other}}\right)\cdot I_{\text{intra}}+P_{N}. \end{array}$$

Based on the semianalytical SINR evolution technique, MUD efficiency is considered here as the percentage of the intracell interference cancelled by the multiuser detector. Thus, by (5), the intracell interference received by base stationconsidering the effect of CBC MUD is

(14) $$\begin{array}{c} I_{\text{total}}\;=\left(1+f_{\text{other}}\right)\cdot \overset{K}{\underset{k=1}{\sum }}\overset{N_{k}}{\underset{\;n_{k}=1}{\sum }}h_{n_{k}}P_{n_{k}}^{*}f\left(\gamma_{k},G_{n_{k}}\right)+P_{N}. \end{array}$$

Based on (10) and (14), we can derive the following expression:

(15) hnkPnk∗(1+WγkRnk) =(1+fother)·∑k=1K∑ nk=1NkhnkPnk∗f(γk,Gnk)+PN =(1+fother)·∑k=1K∑ nk=1Nk11+(W/γkRnk)f(γk,Gnk)Itotal  +PN.

So, we have

(16) hnkPnk∗(1+WγkRnk) ×(1−(1+fother)·∑k=1K∑ nk=1Nk11+(W/γkRnk)f(γk,Gnk))=PN.

According to (16), we can evaluate the optimal power value *P*_*n*_k__*

(17) Pnk∗=(PN)(hnk(1+WγkRnk)    ×(1−(1+fother)·∑k=1K∑ nk=1Nk11+(W/γkRnk)f(γk,Gnk)))−1.

Since each user has power constraint, it is required that the received optimal power *P*_*n*_k__* of user *n*_*k*_ is less than *p*_*n*_*k*__. Thus,

(18) ∑k=1K∑ nk=1Nk11+(W/γkRnk)f(γk,Gnk) ≤1−PNhnk(1+(W/γkRnk))(1+fother)pnk,

for all ∀*n*_*k*_ = 1,…, *N*_*k*_ and ∀*k* = 1,…, *K*.

Define Δ = (*P*_*N*_/(*h*_*n*_*k*__(1 + (*W*/*γ*_*k*_ *R*_*n*_*k*__))(1 + *f*_other_)*p*_*n*_*k*__)); (18) becomes ∑_*k*=1_^*K*^∑_*n*_*k*_=1_^*N*_*k*_^(1/(1 + (*W*/*γ*_*k*_ *R*_*n*_*k*__)))*f*(*γ*_*k*_, *G*_*n*_*k*__) ≤ 1 − Δ.

## 5. Rate Scheduling Strategy

An effective management of rate scheduling depending on the channel quality and the network load situation assigns rate for online users in the cell, and the main rate adjustment is degradation process. However, rate selection strategy is not specified for multiple rates supported in IDMA system.

In this case, a VSG- (variable spreading gain-) IDMA model [15] is selected for the following reasons.

1. VSG model is suitable for the IDMA system. A serious concern in CDMA systems is that the spreading gain is too small to maintain good cross correlation especially for users on condition that data rate is very high. While in IDMA systems, the spreading gain which can realize rate adaptation via the variation of the spreading sequence is irrelevant to distinguish users.
2. VSG model is suitable for satellite system. Rate adaptation in IDMA satellite systems can be simply taken as an adaptive spread gain strategy.

By taking the time-varying property of the satellite mobile communication channel and adaptive transmission technique into account, we can adjust the sending rate dynamically, according to the satellite feedback control and the ARIMI model, to improve the adaptive ability further.

The rate adaptation can be created by the following procedures. When an incoming call arrives to an overloaded cell, the degradation procedure is activated by reducing the service rate of online user in the terrible channel condition. The strategy can ensure different priority levels for different class calls to satisfy well the QoS requirements of various services.

Degradation procedure: the existing calls are degraded according to their priority. Calls of the highest priority get the least consideration for the reduction procedure, whereas calls of low priority can be immediately degraded to a lower bit rate in order to maximize the system capacity. The system assigns the transmission rate, transmitted power, and the only interleaver of the request, to service in the order of voice traffic, video traffic, and background traffic at each frame.

When the resources become overloaded at some frames, background traffic which is pretty relaxed about time delay will firstly reduce transmitted power and the transmission rate of ongoing calls in terrible channel quality. Similarly, if the system is still overloaded, video traffic operates in the same way until degradation factor reaches its maximum value or the system can admit an incoming call. Meanwhile, due to severe restrictions on bit error rate for calls with low priority, the call can be buffered in the system for another access attempt. It not only decreases the blocking probability but also increases the utilization of resource.

In this paper, the basic transmission rate is 15 kb/s and the packets are allowed to transmit at seven rates {15,30,60,120,240,480,960 kb/s} [15]. Correspondingly, the spreading factor *G* ranges from 256 to 4 expressed as 256/2^*k*^  (*k* = 0,1, 2,3, 4,5, 6). In order to analyze conveniently, we use the parameter *w*_*i*,*j*_ (*w*_*i*,*j*_ ∈ {1,2,…, 7}) to indicate the level of degradation in the transmission rate of call *j*in class *i* and it also means the rate adaptation will increase the degradation factor of class *i* by one in progress. Considering the fairness among users, it is unbearable to frequently downgrade rate for the ongoing calls. Thus, degradation factor of class *i* should be lower than allowable degradation limits *w*_*i*_ of class *i*. In other words, degradation factor of class *i* cannot be increased once again unless *w*_*i*,*j*_ < *w*_*i*_.

The transmission rates for multimedia calls can be adjusted to accommodate more calls while satisfying the minimum signal-to-interference ratio (SIR) and transmission rate requirement. So the rate adaptation and power allocation mechanisms can work jointly to decide whether the user should be accepted and assigned with resources.

## 6. Call Admission Control Scheme Based on Channel Quality

In this part, we focus on the CAC algorithm for IDMA systems with various services. In [16], the distinct capacity bottleneck in uplink and the one in downlink are considered, respectively, while both uplink CAC based on interference and downlink CAC based on the base station transmitted power (UD-CAC) are implemented in [9] at the same time. Although the UD-CAC scheme achieves a nice tradeoff between capacity and stability of the system and maximizes the performance of the system, it does not take the effect of MUD into consideration. In [17], the scheme can make accurate estimation of available resource considering the effect of MUD, leading to low outage probability as well as low blocking and dropping probability. However, most of the existing CAC algorithms ignoring the influence of satellite channel cannot adaptively change with dynamic environment. On this basis, a multiservice call admission control strategy based on channel quality is proposed. The proposed scheme which is conjunct with rate scheduling and buffering strategy can guarantee high power efficiency and throughput for multimedia traffic even in heavy load conditions, illustrating the high efficiency of CBC MUD. Especially when communication quality of users get worse, system can take into account the quality of service guarantees, interference and channel quality, and so forth to make judgments on whether it is reasonable and feasible to admit new calls and whether to adjust the ongoing calls to increase access opportunity.

The special concerns in designing the scheme are as follows:

- the satellite resource allocation adaptively changes with dynamic environment and can further solve the global optimization problems;
- the proposed rate adaptation and buffering strategy achieve better performance;
- the traffic asymmetry and distinct capacity bottlenecks between uplink and downlink will be discussed in detail.

**Figure 10:**
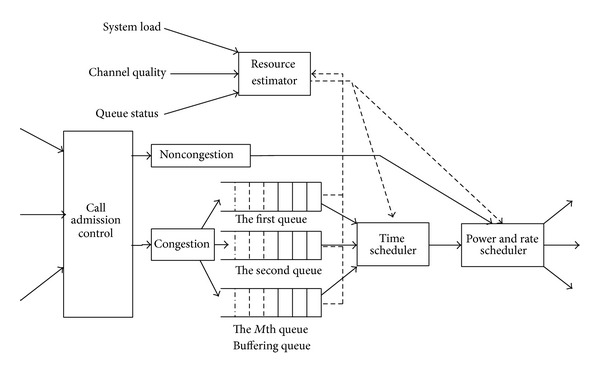
The IDMA MAC protocol based on channel quality.

The CBC MUD scheme and SINR evolution technique for fast performance evaluation of IDMA are briefly introduced in Section 4. Here we extend this accurate and effective technique to the estimation of interference level. The proposed CAC scheme working in conjunction with rate scheduling and buffering strategy is explained in Figure 10. The transmission rates for multimedia calls can be adjusted to accommodate more calls according to different traffic priorities while meeting their minimum signal-to-interference ratio (SIR) and QoS requirement, only when congestion occurs. Also, the buffering strategy is introduced to hold the call which cannot be admitted at once to increase access opportunity according to different delay characteristics of the traffic.

### 6.1. Estimation of Uplink Interference Level

With (11) and (12), we can represent *I*_total_ = (1 + *f*_other_) · *I*_intra_ + *P*_*N*_. Further, assume that the required transmitted power of active user *n*_*k*_ is *S*_*n*_*k*__. Then, *S*_*n*_*k*__ is written as

(19) $$\begin{array}{c} S_{n_{k}}=h_{n_{k}}P_{n_{k}}. \end{array}$$

According to (6), the received bit energy to interference power spectral density ratio for service type *k*, (*E*_*b*_/*I*_0_)_*k*_ can be written as

(20) $$\begin{array}{c} {\left(\frac{E_{b}}{I_{0}}\right)}_{k}=\frac{S_{n_{k}}}{\left(I_{\text{total}}-S_{n_{k}}\right)}\cdot \frac{W}{R_{n_{k}}}\geq \gamma_{k}. \end{array}$$

In order to evaluate the effect of an active user to the system interference, we define the load factor of a single connection as

(21) $$\begin{array}{c} L_{n_{k}}=\frac{S_{n_{k}}}{I_{\text{total}}}=\frac{1}{\left(1+\left(W/\gamma_{k}R_{n_{k}}\right)\right)}. \end{array}$$

Based on the above formulas and the effect of CBC MUD, the total intracell interference power from users in home cell can be written as

(22) $$\begin{array}{c} I_{\text{intra}}=\overset{K}{\underset{k=1}{\sum }}\overset{N_{k}}{\underset{\;n_{k}=1}{\sum }}L_{n_{k}}\cdot I_{\text{total}}f\left(\gamma_{k},G_{n_{k}}\right). \end{array}$$

Similarly, we define the fractional load factor in the home cell *η* as

(23) $$\begin{array}{c} \eta =\left(1+f_{\text{other}}\right)\overset{K}{\underset{k=1}{\sum }}\overset{N_{k}}{\underset{\;n_{k}=1}{\sum }}L_{n_{k}}f\left(\gamma_{k},G_{n_{k}}\right), \end{array}$$

which is normally used as the home cell load indicator [14]. Based on (11) and (22), the total interference received in the home cell can be written as

(24) $$\begin{array}{c} I_{\text{total}}=\eta \cdot I_{\text{total}}+P_{N}=\frac{P_{N}}{1-\eta }. \end{array}$$

With the derivative form of (24), the uplink power increase of the total interference level due to a new requiring user-*n*_*k*_ with CBC MUD in IDMA systems can be estimated as follows:

(25) $$\begin{array}{c} \Delta I=\frac{I_{\text{total}}f\left(\gamma_{k},G_{n_{k}}\right)}{1-\eta -f\left(\gamma_{k},G_{n_{k}}\right)\Delta L}\Delta L. \end{array}$$

### 6.2. Estimation of Downlink Transmitted Power Level

Similar to the uplink, all users share the common bandwidth and each new connection increases the interference level of other connections, affecting the service quality expressed in terms of a certain (*E*_*b*_/*I*_0_)_*i*_^*d*^. For *N* users receiving signals simultaneously from a given cell, the received (*E*_*b*_/*I*_0_)_*i*_^*d*^ can be written as

(26) $$\begin{array}{c} {\left(\frac{E_{b}}{I_{0}}\right)}_{i}^{d}=\frac{W}{{R_{i}}^{d}}\cdot \frac{g_{i0}p_{i}^{N}}{\sum_{\begin{array}{c} j=1\\ j\neq i \end{array}}^{N}\theta g_{i0}p_{j}^{N}+g_{i0}P_{p}+I_{i}+P_{N}}\geq {\gamma_{i}}^{d},\\ P_{\text{total\_}N}=\overset{N}{\underset{j=1}{\sum }}p_{j}^{N}+P_{p}, \end{array}$$

where *P*_total_*N*_ represents the base station transmitted power, *p*_*j*_^*N*^  (*j* = 1,2,…, *N*) is the power devoted to the *j*th user, *I*_*i*_ is the intercell interference observed by the *i*th user, *g*_*i*0_ is the path loss to the user *i*, *P*_*P*_ is the power assigned to pilot channel, and *θ* ∈ (0,1] is the orthogonality factor in the downlink direction. The minimum transmitted power *p*_*i*_^*N*^ satisfying the *i*th user demands can be expressed as

(27) $$\begin{array}{c} p_{i}^{N}=L_{i}^{d}\left(\theta \overset{N}{\underset{j=1}{\sum }}p_{j}^{N}+P_{p}+\frac{I_{i}+P_{N}}{g_{i0}}\right),\\ L_{i}^{d}=\frac{{\gamma_{i}}^{d}{R_{i}}^{d}}{W+\theta {\gamma_{i}}^{d}{R_{i}}^{d}}. \end{array}$$

The latter expression is commonly known as the downlink load factor for the *i*th user.

Assume that the subscript number of a new call is 0; the total amount of the users in home cell is *N* + 1 if it is accepted. Now the transmitted powerto the *i*th user is

(28) $$\begin{array}{c} p_{i}^{N+1}=L_{i}^{d}\left(\theta \overset{N}{\underset{j=0}{\sum }}p_{j}^{N+1}+P_{p}+\frac{I_{i}+P_{N}}{g_{i0}}\right). \end{array}$$

From the above, the increase in power demand to the *i*th user Δ*p*_*i*_ is estimated as follows:

(29) $$\begin{array}{c} \Delta p_{i}=L_{i}^{d}\left(\theta \overset{N}{\underset{j=1}{\sum }}\Delta p_{j}+\theta p_{0}\right), \end{array}$$

and transmitted power to the 0th user can be written as

(30) $$\begin{array}{c} p_{0}=L_{0}^{d}\left(\theta \overset{N}{\underset{j=1}{\sum }}p_{j}^{N}+P_{p}+\frac{I_{0}+P_{N}}{g_{00}}\right)\frac{\left(1-\theta \sum_{j=1}^{N}L_{j}^{d}\right)}{\left(1-\theta \sum_{j=0}^{N}L_{j}^{d}\right)}. \end{array}$$

After accumulating all the Δ*p*_*j*_  (*j* = 1,…, *N*), total transmitted power for *N* + 1 users is

(31) Ptotal_N+1=Ptotal_N+∑j≠0Δpj+p0=L0dPtotal_N(θ+((I0+PN)/(g00Ptotal_N)))(1−θ∑j=0NLjd) +Ptotal_N,

where ∑_*j*=0_^*N*^ *L*_*j*_^*d*^ is the downlink fractional load factor.

Similar to the uplink, *f*_other_^*d*^ is defined as the ratio of the total base station transmitted power from adjacent cells to intracells, and the power generated by base station in the home cell is set as its typical value which is 0.55 [18]. When the background noise is ignored, (31) can be written as

(32) $$\begin{array}{c} P_{\text{total}\_N+1}=\frac{L_{0}^{d}P_{\text{total}\_N}\left(\theta +f_{\text{other}}^{d}\right)}{\left(1-\theta \sum_{j=0}^{N}L_{j}^{d}\right)}+P_{\text{total}\_N}. \end{array}$$

Considering that the downlink receivers in IDMA systems also benefit from CBC detection [10], the uncancelled percentage of intracell interference is *f*(SINR). Then the orthogonality factor in the downlink direction can be equivalent to *f*(SINR). With the aid of SINR evolution, the downlink load factor for the *i*th user and the total transmitted power can be accurately and easily estimated as

(33) $$\begin{array}{c} L_{i}^{d}=\frac{{\gamma_{i}}^{d}{R_{i}}^{d}}{W+f\left(\text{SIN}{\text{R}}_{i}\right){\gamma_{i}}^{d}{R_{i}}^{d}},\\ P_{\text{total}\_N+1}=\frac{L_{0}^{d}P_{\text{total}\_N}\left(f\left(\text{SIN}{\text{R}}_{0}\right)+f_{\text{other}}\right)}{\left(1-\sum_{j=0}^{N}f\left(\text{SIN}{\text{R}}_{j}\right)L_{j}^{d}\right)}+P_{\text{total}\_N}. \end{array}$$

### 6.3. The Proposed Admission Control Algorithm

**Figure 11:**
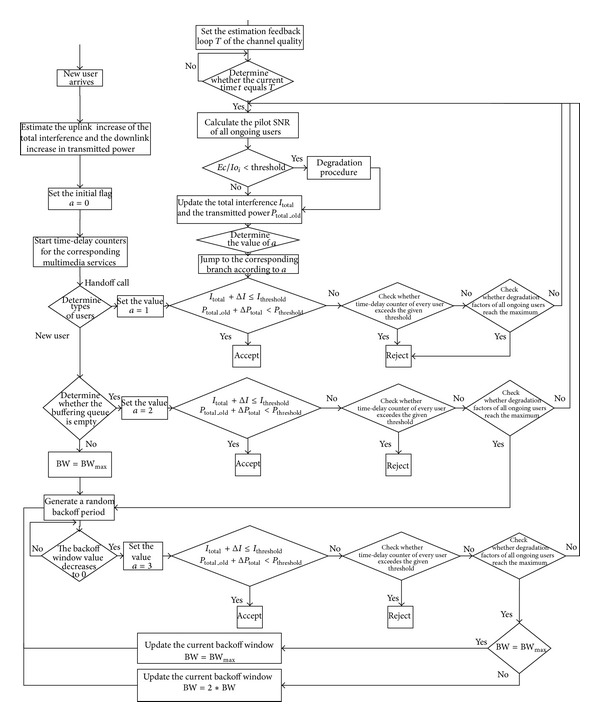
Flowchart of the proposed CAC scheme.

According to UD-CAC scheme [9], determination conditions of uplink CAC based on interference and downlink CAC based on the base station transmitted power (UD-CAC) are *I*_total_old_ + Δ*I* ≤ *I*_THRESHOLD_ and *P*_total_old_ + Δ*P*_total_ < *P*_threshold_. Based on feedback interval of channel quality, predicted values of channel quality *γ* ^*i*^ for the user *i* apart from those of voice traffic are compared with the target SNR_*i*_. The proposed CAC scheme consists of seven stages, explained in Figure 11. Follow those steps according to the priority of different services.

*Stage 1.* Channel quality detection.

According to the service requirements (such as transmission rate and activating factor) of the call, determine whether or not *γ* ^*i*^ > *γ*_req_^*i*^. If yes, no further operations are performed; if not, start the rate adaptation. 

*Stage 2.* Rate adaptation algorithm.

Plus one for the user's downgrade factor *w*_*i*,*j*_ and update the current transmission rate and interference factor. Meanwhile, judge whether degradation factor of class *i* is lower than allowable degradation limits *w*_*i*_. If not, do nothing; else, repeat* Stage  1*. 

*Stage 3.* Resource estimation and forecast period.

For uplink, we estimate the total interference *I*_total_ for the current system and incremental interference Δ*I* after accepting new users. And for downlink, the total transmitted power from the base station *P*_total_old_ and the expected increase in transmitted power Δ*P*_total_ should be estimated.

When a handoff call arrives, it will be accepted if

(34) Itotal_old+ΔI≤Ithreshold,Ptotalold+ΔPtotal<Pthreshold

is satisfied. Else, go to* Stage 4*. But for a new call, determine whether the buffering queue is empty. If not, go to* Stage 5*; else, decide whether to admit the call according to

(35) Itotal_old+ΔI≤ITHRESHOLD,Ptotalold+ΔPtotal<Pthreshold.

If the above equation is workable, the call is accepted. Otherwise, step to* Stage 6*.

*Stage 4.* Degradation procedure for handoff user.

In accordance with class priorities in ascending order, the ongoing calls with poor channel quality perform degradation process. When degradation factors of all ongoing users (excluding voice services) have reached the maximum and (34) still does not valid, then the handoff call will be refused.

*Stage 5.* Implementation of buffering strategy (see the part* D*).

*Stage 6.* Degradation procedure for new user.

Similarly, according to class priorities in ascending order, the ongoing calls with poor channel quality perform degradation process. But unlike the processes for handoff call, when degradable rate of every class has been exhausted and (35) still does not valid, then put the new call in cache queue to be detected when the backoff window value decreases to 0.

*Stage 7.* When the call is completed, release all resources and update the available capacity in the system at the same time.

### 6.4. Buffering Strategy

When a new call requests the access to the system, test whether the delay queue is empty and when the delay queue is nonempty, enforce buffering strategy. Due to the necessity for satisfying the different requirements of various services for delay time, time-delay counters for multimedia services designed with different threshold values should judge in real time whether the cumulative delay exceeds a threshold. If so, reject the call; if not, generate a random backoff period. The measurements for random delay time are divided discretely in timeslot *τ*.

The initial settings for the maximum and minimum backoff window value are BW_max⁡_ and BW_min⁡_, respectively. Specific steps are as follows.

*Stage 1.* Depending on the type of new call and the backoff window in the request packet, the backoff window is set as the minimum value BW = BW_min⁡_. 

*Stage 2.* Random delay time *T* is the product of the timeslot and the random backoff window value BW_now_ ranging from 0 to BW, which is *T* = BW_now_ · *τ*. 

*Stage 3.* The initial setting for the current backoff window value is BW_now_, and reset the backoff window counter. 

*Stage 4.* When the backoff window value gradually decreases to 0, check whether the condition (35) is satisfied. If so, the call is accepted; if not, execute a new rate scheduling immediately and check whether the condition (35) is satisfied. If so, the call is also accepted. Otherwise, step to next stage. 

*Stage 5.* Check whether time-delay counter of every user exceeds the given threshold. If satisfied, reject; otherwise, go to next stage. 

*Stage 6.* If BW_now_ ≥ BW_max⁡_, update the current backoff window BW = BW_min⁡_; otherwise, update the current backoff window BW = 2∗BW_min⁡_. Then, continue to* Stage 3*.

## 7. Performance Evaluation

### 7.1. Traffic Model and Simulation Parameters

A 37-cell layout is considered, in which mobiles are distributed uniformly. Suppose the uplink and downlink bandwidth are 3.84 MHz. For each cell there are three classes of traffic, that is, the class of conversational, streaming, and interactive. Conversational class is constant bit rate (CBR) under an ON/OFF activity model. As in [18], streaming class is modeled as a discrete state, continuous-time Markov process. The interactive traffic is approximately modeled as the Pareto process. Further, there are two major types of calls which can arrive at any cell: new calls originated from the local cell and handoff calls coming from adjacent cells corresponding to each traffic. Since it is more reluctant to block a handoff call than a new call, the handoff calls should be given a higher priority. Based on the multimedia traffic requirements, the traffic characteristics and QoS requirements are defined in Table 1.

### 7.2. Simulation Results and Performance Evaluation

The paper analyzes several parameters for ease of comparison: the blocking probability of new calls, the dropping probability of handover calls, outage probability, throughput, package loss, and average delays (waiting time in the queue to start transmitting).

**Figure 12:**
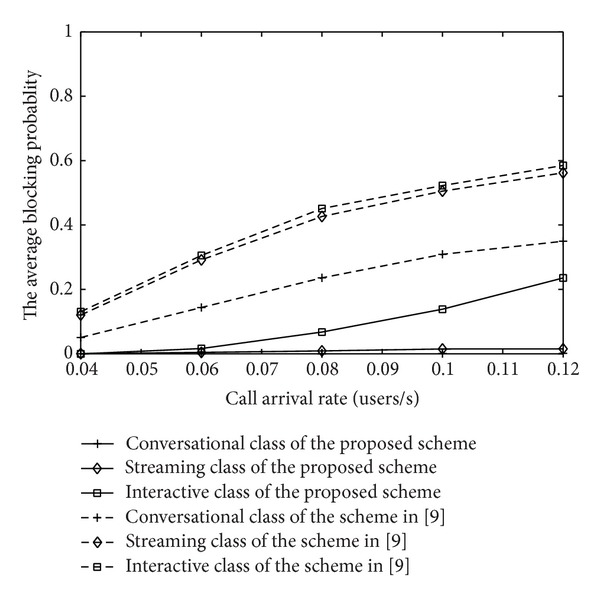
The blocking probability of IDMA system.

**Figure 13:**
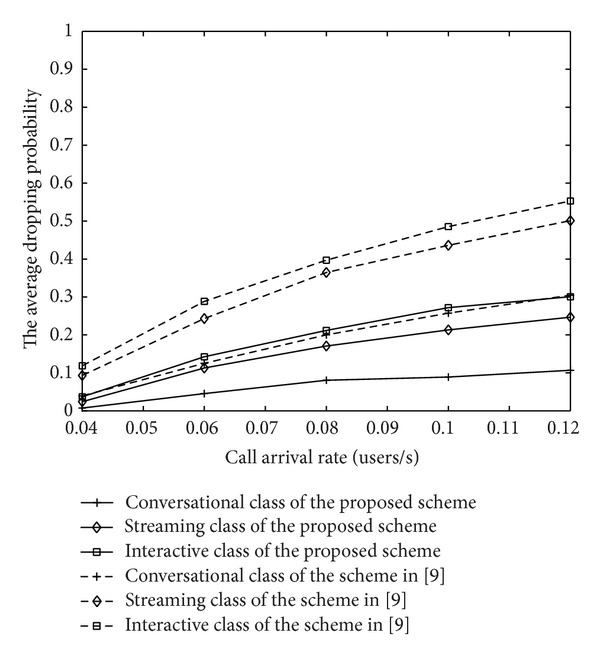
The dropping probability of IDMA system.

With the call arrival rate varying, the blocking probability of new calls and the dropping probability of handover calls under two strategies and different classes are shown in Figures 12 and 13, respectively. We could see that the higher the priority level is, the lower the average blocking probability and the dropping probability are. Meanwhile, due to rate adaptation and buffering policy, the proposed MAC strategy based on the channel quality greatly reduces the average blocking probability and the dropping probability. Performance of proposed algorithm is obviously much more excellent than the scheme in [9], which guarantees the priority and fairness between new calls and handoff calls.

**Figure 14:**
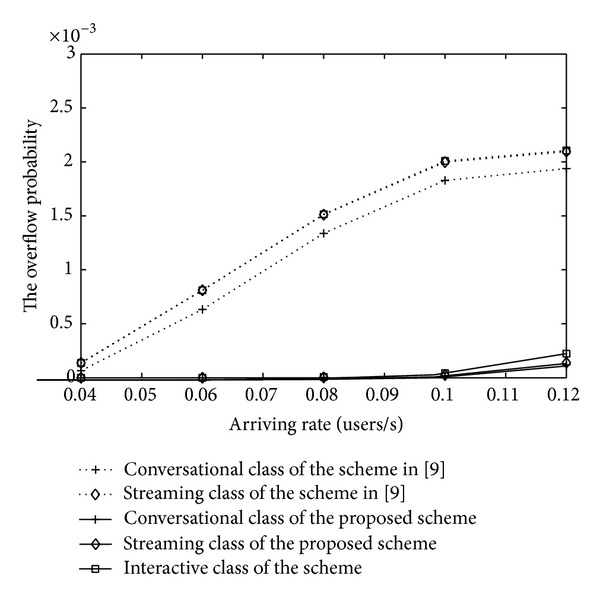
The overflow probability of IDMA system.

In order to verify the ability of the proposed scheme to assure the QoS of all users during their whole service time, the schemes are assessed in terms of outage probability.  Figure 14 illustrates the changing of the outage probability with the arriving rate. As is shown, the outage probability is relevant to the priority of various classes and the arriving rate. What is more, the proposed MAC strategy based on the channel quality adopts the rate degradation to handle blocking problems, which reduce the link load factor and the whole interference. At the same time, buffering strategies can balance the traffic load effectively. The proposed scheme cannot only ensure a low average blocking probability as well as a low dropping probability but also ensure an ideal overflow probability of the IDMA system.

**Figure 15:**
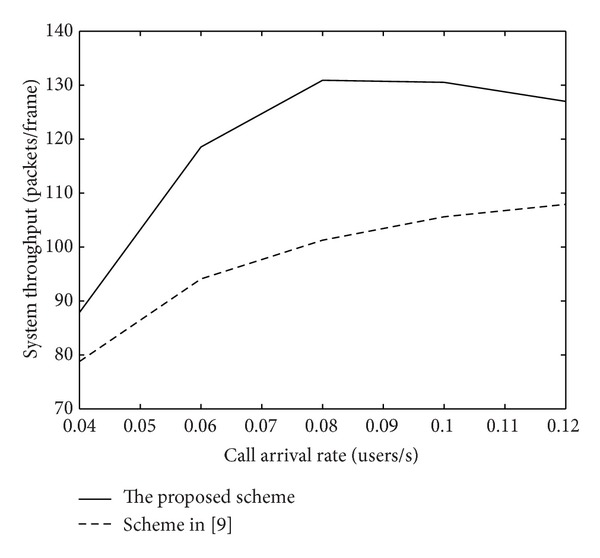
System throughput.

Figure 15 shows the resource utilization in the condition of different traffic loads. The throughput is defined as the total bit rate that system can maintain. We can observe that the throughput increases with the increment of network load and starts declining when arriving rate exceeds a point. For the proposed MAC strategy, the throughput is higher than the one in the scheme in [9] when congestion occurs, most users are accepted by means of buffering strategy, thus guaranteeing QoS and ensuring the fairness of each service. In accordance with simulation testing, the proposed MAC strategy can relax the load in the network effectively and improve the whole system quality for the communication to some extent.

**Figure 16:**
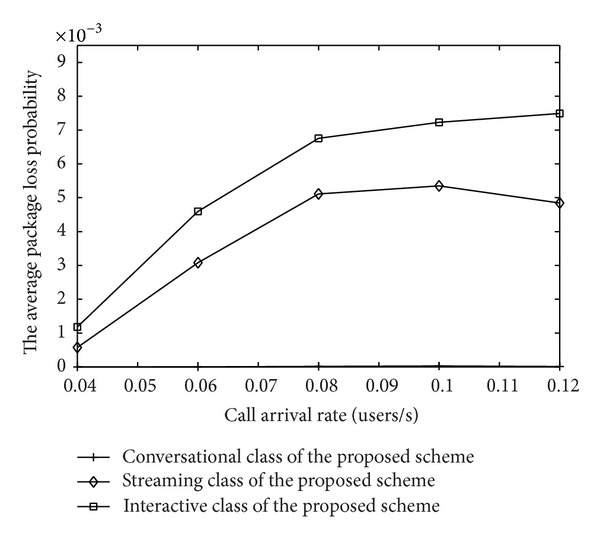
The package loss of IDMA system.

The package losses of different priorities are compared in Figure 16. When congestion occurs, the call can be buffered in the system for another access attempt. However, when the backoff window length is too large, unpredictable delay leads to the packet loss unavoidably. In addition, rate adaptation, that the existing calls are degraded according to their priority in order to accept more session class will lead to increasing the rate of the losing packet. Therefore, relative to strategy in [9], the proposed MAC strategy based on the channel quality sacrifices parts of the transmission rate to ensure the better performance.

**Figure 17:**
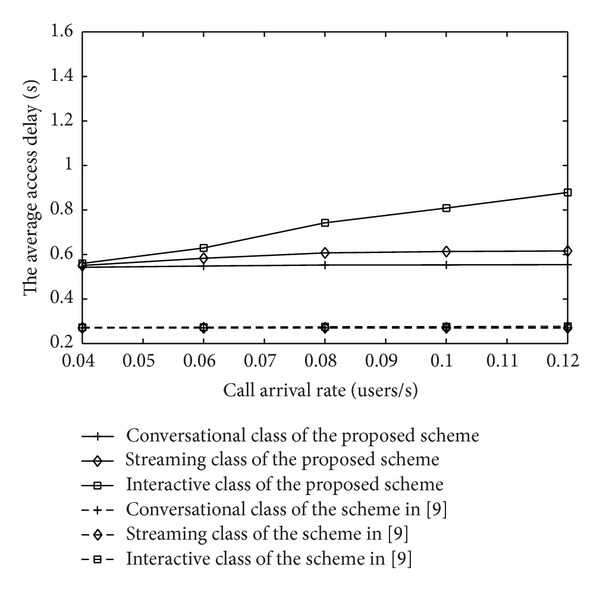
The average end-to-end delay of IDMA system.

The average end-to-end delay is another important factor of system evaluation. The influence of an improved schedule strategy on the average end-to-end delay is simulated in Figure 17. As is shown in Figure 17, the average end-to-end delay of the proposed MAC strategy is greater than that of scheme in [9]. Here we focus on the waiting time including access delay that is the time cost in sending an access request successfully and the queuing time that the messages spend in the buffer. With the proposed MAC, the unaccepted call would cache in a delay queue temporarily and this increases the waiting time compared with the scheme in [9] when considering the same access delay.

## 8. Conclusions

In this paper, we derived the minimum-power allocation algorithm for mobile terminals that transmit multimedia traffic in wide-band IDMA system. We also proved that prediction of satellite channel quality is requisite of meeting dynamically changing environments. With effective power control and accurate channel information per timeslot, we proposed a new rate adaptation for the MAC protocol of IDMA wireless system. Furthermore, to enhance the performance of the MAC protocol, we developed a new CAC algorithm based on minimum-power allocation as well as rate scheduling and buffering strategies. The new MAC protocol can be adaptive to guarantee the QoS requirements of all kinds of services, improving the fairness and the utilization of resources efficiency. In summary, IDMA-based MAC protocol with consideration on channel quality is a promising protocol for satellite networks.

## Figures and Tables

**Table 1:** Traffic model.

	Conversational class		Streaming class		Interactive class	
Link	Uplink	Downlink	Uplink	Downlink	Uplink	Downlink
Activity factor	0.6	0.6	0.00285	0.95	0.00285	0.015
Data rate/(kbit/s)	15		{15,30,60,120,240,480,960}		{15,30,60,120,240,480,960}	
*E*_*b*_/*N*_0_ target/dB	7		5		3.7	
Portion of arrival						
New call	30%		5%		15%	
Handoff	30%		5%		15%	
Mean call duration/s	100		1200		1200	
Priority	Premium		Assured		Best effort	
Max delay for new calls/frame calls	2		7		20	
*w*_*i*_	1		3		6	
